# Hormone Resistance in Two MCF-7 Breast Cancer Cell Lines is Associated with Reduced mTOR Signaling, Decreased Glycolysis, and Increased Sensitivity to Cytotoxic Drugs

**DOI:** 10.3389/fonc.2014.00221

**Published:** 2014-09-03

**Authors:** Euphemia Yee Leung, Ji Eun Kim, Marjan Askarian-Amiri, Wayne R. Joseph, Mark J. McKeage, Bruce C. Baguley

**Affiliations:** ^1^Auckland Cancer Society Research Centre, University of Auckland, Auckland, New Zealand

**Keywords:** breast cancer cells, PI3K, mTOR, estrogen receptor, cytotoxic drugs, cisplatin, tamoxifen

## Abstract

The mTOR pathway is a key regulator of multiple cellular signaling pathways and is a potential target for therapy. We have previously developed two hormone-resistant sub-lines of the MCF-7 human breast cancer line, designated TamC3 and TamR3, which were characterized by reduced mTOR signaling, reduced cell volume, and resistance to mTOR inhibition. Here, we show that these lines exhibit increased sensitivity to carboplatin, oxaliplatin, 5-fluorouracil, camptothecin, doxorubicin, paclitaxel, docetaxel, and hydrogen peroxide. The mechanisms underlying these changes have not yet been characterized but may include a shift from glycolysis to mitochondrial respiration. If this phenotype is found in clinical hormone-resistant breast cancers, conventional cytotoxic therapy may be a preferred option for treatment.

## Introduction

The mTOR pathway is a key regulator of multiple cell signaling pathways, integrating growth factors, nutrients, energy, and stress (1). In breast cancer cells, mTOR signaling is linked through phosphatidylinositol 3-kinase (PI3K) and Akt/protein kinase B (PKB) (2) to signaling from external cellular receptors such as EGFR. Increased signaling through the mTOR pathway has been proposed to control distinct regulatory motifs that promote a pro-invasion translational program (3, 4) and to control important mechanisms for endocrine resistance (5). In the MCF-7 breast cancer cell line, mTOR activity is thought to be responsible for the constitutive activity of Akt, and inhibition of mTOR activity restores response to the antiestrogen tamoxifen (6). It was therefore surprising to find, during the course of selecting cultures of MCF-7 cells for resistance to tamoxifen or to estrogen deprivation, two cell lines (TamC3 and TamR3, respectively) that had reduced mTOR signaling, as shown by reduced phosphorylation of the downstream enzymes Akt/PKB and p70S6K, as well as increased resistance to the mTOR inhibitors rapamycin and everolimus (7, 8) and to the dual PI3K/mTOR inhibitors NVP-BEZ235 and GSK2126458 (9). The TamC3 and TamR3 resistant cell lines were distinguished from the parental line by having reduced cellular DNA contents (ploidies), strongly reduced modal cell volumes, slightly reduced cell cycle times, and altered signaling pathway usage (7). The differing ploidies suggest that they arose from expansion of minor subpopulations of the original MCF-7 cell line rather from metabolic adaptation of the parental line. The TamC3 and TamR3 sub-lines also showed increased expression of ER, progesterone receptors (PR), and epidermal growth factor receptor-2 (EGFR2; HER2) (7, 9, 10). Here, we have investigated the properties of these two cell lines that might explain their behavior. We have measured their sensitivity, in comparison to that of the parental line, to DNA damaging anticancer drugs, mitotic poisons, or oxidative damage. We have also determined whether the phenotype is associated with decreased glycolysis and increased mitochondrial respiration.

## Materials and Methods

### Cell culture

**Figure 1:**
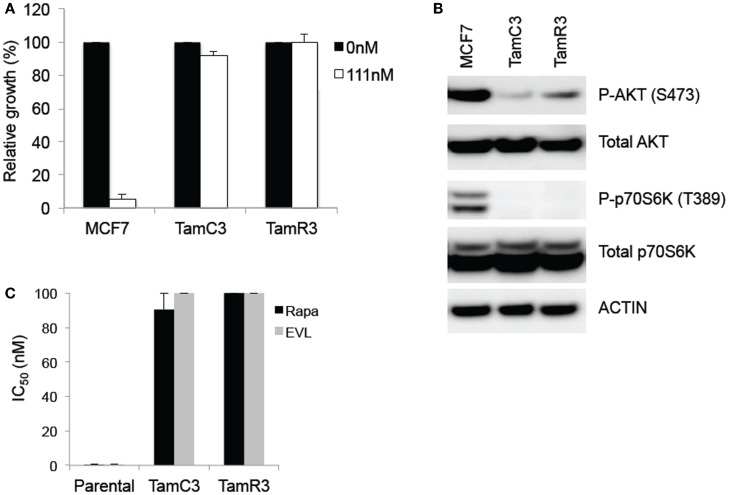
**Comparison of basal levels of Akt- and p70S6K-phosphorylation in the MCF-7 line and its sub-lines and drug sensitivity to 4-hydroxytamoxifen and mTOR inhibitors**. **(A)** Effects of 4-hydroxytamoxifen on the proliferation of MCF-7 parental, TamC3, and TamR3 cell lines. The cell lines were exposed to 4-hydroxytamoxifen (111 nM) for 4 days and cell proliferation was measured by a thymidine incorporation assay. *Significantly different from MCF-7 parental line (Holm–Sidak test; *p* < 0.05). **(B)** Relative expression of phosphorylated p70S6K and Akt, and total p70S6K and Akt in MCF-7, TamC3, and TamR3 cell lines. Actin is shown as a loading control. **(C)** IC_50_ values for rapamycin and everolimus in MCF-7 parental, TamC3, and TamR3. Cells were treated with rapamycin or everolimus for 4 days with [^3^H]-thymidine added for the last 6 h. The highest drug concentration is depicted where 50% growth inhibition was not reached. Bars indicate SE (duplicate experiments).

Culture conditions have been described previously (7); MCF-7 was purchased from the American Type Culture Collection (ATCC). The TamR3 cell line was generated by growth of MCF-7 cells in phenol-red-free RPMI containing 10% charcoal-stripped fetal bovine serum (Invitrogen, Auckland, New Zealand), over a period of 3 months to progressively increasing concentrations of tamoxifen (1 nM to 1 μM in ethanol) and then maintaining them for >15 months in 1 μM tamoxifen. The TamC3 cell line was generated by exposure of MCF-7 cells for >16 months to the above growth medium but lacking tamoxifen. Both TamC3 and TamR3 cell lines are cross-resistant to 4-hydroxytamoxifen (Figure 1A). All experiments were carried out on cells grown in their respective growth media but without tamoxifen.

### Chemicals and reagents

Propidium iodide, tamoxifen, camptothecin, doxorubicin, hydrogen peroxide, cisplatin, carboplatin, oxaliplatin, paclitaxel, docetaxel, fluorouracil, bicinchoninic acid (BCA), 4-hydroxytamoxifen, and tamoxifen were from Sigma (Auckland, New Zealand).

### Measurement of DNA content for cell cycle analysis

Cells (1 × 10^6^ cells) were grown in six-well plates and incubated with inhibitors for 24 h. Cells were harvested, washed with 1% FCS/phosphate-buffered saline (PBS), resuspended in 200 μl of PBS, fixed in 2 ml of ice-cold 100% ethanol, and stored overnight at −20°C. The cells were washed and resuspended in 1 ml of 3% FCS/PBS containing RNase (1 μg/ml) and propidium iodide (PI) (10 μg/ml) for 30 min at room temperature. DNA content was determined using forward scatter (FSC) intensity by PI staining based on a total 30,000 acquired events by FACScan cytometry.

### Measurement of DNA damage

DNA damage was measured after harvesting cells, washing and resuspended in 200 μl of PBS, fixing in 2 ml of ice-cold 100% ethanol, and storing overnight at −20°C. Cells were resuspended in 1 ml of blocking buffer (1% FCS/PBS), and incubated with antibody to γ-H2AX (phosphorylated Ser139) (Millipore, USA) in blocking buffer (1:500 dilution) at room temperature for 2 h. Cells were washed, incubated with goat anti-mouse Alex 488 Fab fragment secondary antibody (Invitrogen, New Zealand) (1:400 in blocking buffer for 1 h, at room temperature; dark), washed and resuspended in 1 ml of blocking buffer containing RNase (1 μg/ml) and propidium iodide (PI) (10 μg/ml) for 30 min at room temperature. Cells were analyzed in a Becton Dickinson LSRII and profiles were analyzed with ModFit LT 3 software.

### Cell proliferation assay

Cell proliferation was measured using a thymidine incorporation assay in which 3,000 cells were seeded in 96-well plates in the presence of varying concentrations of drugs for the indicated number of days. Cell proliferation was measured either by thymidine uptake assay (for 4-hydroxytamoxifen, everolimus, and rapamycin) or sulforhodamine B colorimetric assay (for all other inhibitors). Briefly, [^3^H] thymidine (0.04 μCi) was added to each well and plates were incubated for 5 h, after which the cells were harvested onto glass–fiber filters using an automated TomTec harvester. Filters were incubated with Betaplate Scint and thymidine incorporation was measured in a Trilux/Betaplate counter. Cell proliferation was determined by the percentage incorporation of [^3^H]thymidine. All experiments were carried out in triplicate, and repeated at least twice. The sulforhodamine B colorimetric assay, which is based on the measurement of cellular protein content, was used to measure cell density (11). After drug treatment for 3 days, cells were fixed with 10% (wt/vol) trichloroacetic acid and stained for 30 min, and the excess dye was removed by washing repeatedly with 1% (vol/vol) acetic acid. The protein-bound dye was dissolved in Tris base solution (10 mM) for optical density determination at 510 nm using a microplate reader. All experiments were done in triplicate, and were repeated at least twice. Optimal cell densities were previously determined to select initial cell densities that ensured that cells were in logarithmic phase for the experiments.

### Glucose uptake

Cells (1 × 10^6^ cells) were grown in six-well plates and incubated for 24 h. Cells were incubated with 1 μCi (1 μl) of 2-deoxy-d-[1-^3^H]glucose (Perkin Elmer) in 1 ml of media for 20 min at 37°C. The cells were washed two times with ice-cold PBS, and lysed in 1 ml of 1% SDS. Cell lysate 0.8 ml in 4.5 ml Ecoscint H (National Diagnostics LS-275) was counted on a scintillation device for 1 min. All experiments were done in triplicates wells, and repeated at least two times.

### Measurement of reactive oxygen species

Intracellular reactive oxygen species (ROS) were detected with the cell permeable fluorescent probe 5-(and 6-)-chloromethyl-2′,7′-dichlorodihydrofluorescein diacetate (CM-H_2_DCFDA) (Life Technologies). Cells (1 × 10^6^) were grown in six-well plates and incubated for 24 h. Cells were incubated with 10 μM CM-H_2_DCFDA in PBS with 2% FCS for 30 min in the dark at 37°C. Cells were then trypsinized and analyzed in a Becton Dickinson LSRII and data were analyzed with FlowJo software. All experiments were done in 10 wells, and repeated at least two times.

### Measurement of mitochondrial reductive activity

Mitochondria activity is measured by Alamar Blue assay (Life Technologies). Superoxide dismutase (SOD) was measured using a water-soluble tetrazolium salt (WST-1) (purchased from Roche) (12). The stable tetrazolium salt WST-1 is cleaved to a soluble formazan by a complex cellular mechanism that occurs primarily at the cell surface. This bioreduction is largely dependent on the glycolytic production of NAD(P)H in viable cells. Therefore, the amount of formazan dye formed directly correlates to the number of metabolically active cells in the culture. All experiments were done in 10 wells, and repeated at least two times.

### Measurement of l-lactate

Cells (1 × 10^5^) were seeded the previous day, the culture medium was replaced with 500 μl growth medium and the cells were grown for 4 h at 37°C. Medium was removed from cells and lactate levels in the extracellular medium were measured using the l-lactate Colorimetric Assay Kit (Abcam, Cambridge, MA, USA). Lactate levels were normalized to final cell counts. All experiments were done in five wells, and repeated at least two times.

### Measurement of cisplatin uptake

Uptake of cisplatin by MCF-7 parental and sub-lines utilized inductively coupled plasma spectrometry (ICP-MS; carried out at LabPLUS, Auckland, New Zealand). Cells were incubated in transport buffer (125 mM NaCl, 4.8 mM KCl, 5.6 mM d-glucose, 1.2 mM CaCl_2_, 1.2 mM KH_2_PO_4_, 1.2 mM MgSO_4_, and 25 mM HEPES pH 7.4) containing cisplatin (30 μM) for 30 min at 37°C in 5% CO_2_. Following incubation, cells were washed once with ice-cold PBS, twice with PBS containing 10 mM EDTA, and lysed in 70% nitric acid at room temperature for at least 2 h. Cellular protein content was quantified using previously described method (13). All experiments were done in duplicate wells, and repeated at least two times.

### Mammosphere formation

MCF-7 parental and sub-lines were trypsinized from the monolayer culture and cell suspensions were then seeded in 96-well non-tissue culture treated plates, with 1,000 cells per well in 8 replicates per experiment. Cells were grown in MammoCult^®^ medium (Stem Cell Technologies), and were kept at 37°C with 5% CO_2_. Mammospheres were counted after 7 days. All experiments were repeated at least two times.

### Immunoblotting

Parental MCF-7 cells and the variants TamC3 and TamR3 were grown to logarithmic phase, washed twice with ice-cold PBS and lysed in SDS lysis buffer according to the manufacturer’s protocol (Cell Signaling Technology, Danvers, MA, USA). Protein concentration was quantified using the BCA protein assay reagent bicinchoninic acid (Sigma). Cell lysates containing 20 μg of protein were separated by SDS-PAGE gel electrophoresis and transferred to PVDF membranes (Millipore). Membranes were immunoblotted with antibodies against phospho-Akt (S473), total Akt, phospho-70S6K (T389), total p70S6K (all from Cell Signaling Technology), and actin (Millipore), using SuperSignal West Pico (Thermo Scientific, Waltham, MA, USA) or ECL advance (GE Healthcare, Auckland, New Zealand). Antibody reactivity was visualized using the chemiluminescence detection system by Fujifilm Las-3000. Densitometry was performed using ImageJ. The relative intensity of phosphorylated proteins was normalized using actin as the standard. The fold change was then calculated using MCF-7 as standard reference.

### Statistical analysis

Data were analyzed using a one-way ANOVA.

## Results

### Reduced mTOR pathway signaling and sensitivity to mTOR inhibitors

The TamC3 and TamR3 lines showed increased resistance, in comparison to the parental line, to 4-hydroxytamoxifen (Figure 1A). They also showed reduced phosphorylation of the downstream enzymes Akt/PKB and p70S6K (Figure 1B) and reduced sensitivity to mTOR inhibitors rapamycin and everolimus (Figure 1C).

### Comparison of sensitivity to therapeutic agents

**Figure 2:**
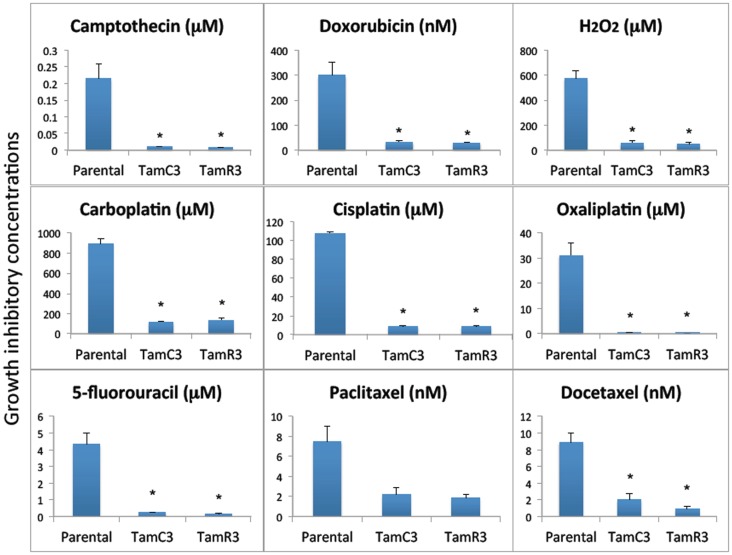
**Growth inhibitory concentrations for MCF-7 and its sub-lines exposed to different drugs IC_30_ values (30% inhibition of growth) are shown for experiments for taxanes and 5-fluorouracil where 50% growth inhibition was not reached, and IC_50_ (50% inhibition of growth) for all of the other agents**. Cells were treated with drugs for 3 days and cell proliferation was measured by the sulforhodamine B assay. Bars indicate SE in two independent experiments. *Significantly different from MCF-7 parental line (*p* < 0.05).

**Figure 3:**
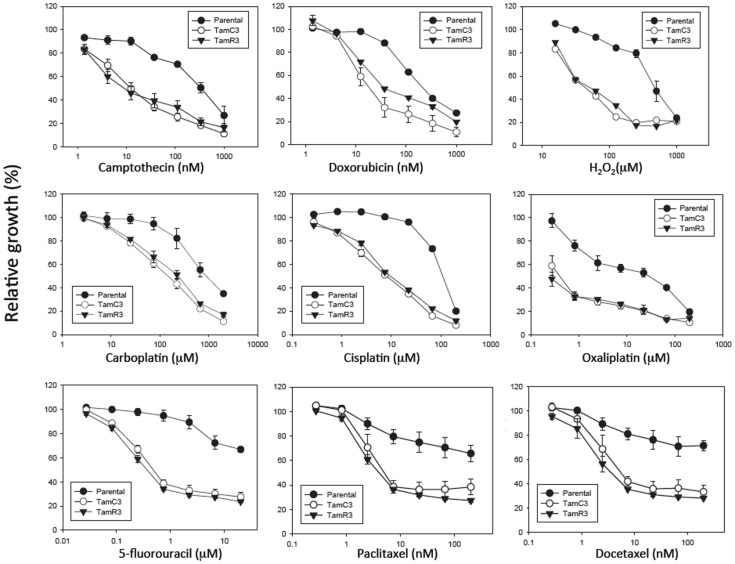
**Growth inhibitory effect of drugs on MCF-7 and its sub-lines**. Cells were treated with drugs for 3 days and cell proliferation was measured by the sulforhodamine B assay. Bars indicate SE in two independent experiments. *Significantly different from MCF-7 parental line (*p* < 0.05).

The sensitivity of MCF-7, TamC3, and TamR3 to a range of chemotherapeutic agents was tested (Figures 2 and 3). These agents included the topoisomerase I inhibitor camptothecin, the topoisomerase II inhibitor doxorubicin, the DNA intrastrand cross-linkers cisplatin, carboplatin, and oxaliplatin, the mitotic inhibitors paclitaxel and docetaxel, and the thymidylate synthase inhibitor 5-fluorouracil. Sensitivity to the oxidizing agent sodium peroxide as also measured. The increased sensitivities of TamC3 and TamR3, in comparison to the parental line, as measured by IC_50_ ratios parental/TamC3 and parental/TamR3, were, respectively, 19- and 29-fold for camptothecin, 9- and 10-fold for doxorubicin, 12- and 12-fold for cisplatin, 7.8- and 6.6-fold for carboplatin, 78- and 183-fold for oxaliplatin, 18- and 27-fold for 5-fluorouracil, 3.5- and 4-fold for paclitaxel, 4.4- and 9.4-fold for docetaxel, and 10- and 11-fold for hydrogen peroxide.

### Comparison of induction of DNA damage by cisplatin and oxaliplatin

**Figure 4:**
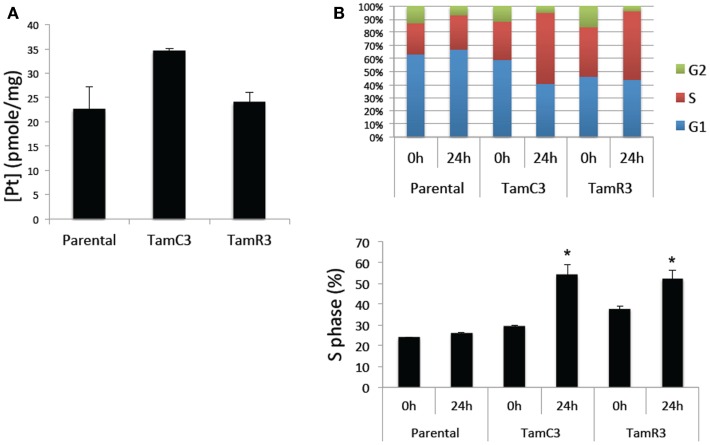
**Cisplatin uptake and S-phase cell cycle arrest in the MCF-7 and its sub-lines**. **(A)** Comparison of cisplatin uptake by MCF-7 parental and its sub-lines. **(B)** Cell cycle proportions (upper panel) in S-phase (lower panel) of MCF-7 cell lines treated with 50 μM cisplatin for 24 h analyzed by flow cytometry. Results were averaged from duplicate samples from one of two independent experiments. *Significant difference from treatment control (*p* < 0.05).

**Figure 5:**
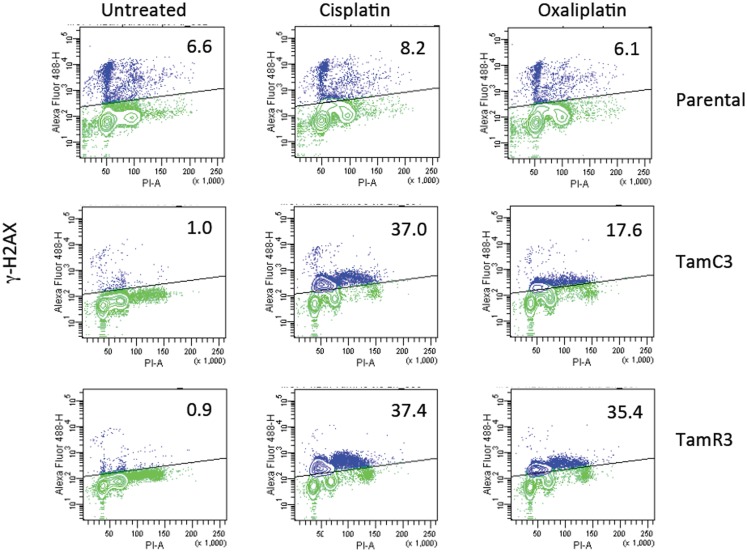
**Induction of DNA damage in the MCF-7 and its sub-lines**. Induction of γ-H2AX phosphorylation following exposure to cisplatin or oxaliplatin for 2 h. MCF-7 parental and derived TamC3 and TamR3 sub-lines were either untreated or exposed cisplatin (50 μM) or oxaliplatin (50 μM) then fixed, stained, and assessed by flow cytometry for γ-H2AX staining. Cellular immunofluorescence (anti-γ-H2AX antibody; *y*-axis) is plotted against DNA content (propidium iodide staining; *x*-axis). The upper half (marked) represents high γ-H2AX phosphorylation and the proportion of the total is indicated. Representative images of two independent experiments are shown.

The increased sensitivity of TamC3 and TamR3 to cisplatin was further investigated. Since resistance has been associated with decreased cellular uptake or increased drug efflux (14), we measured cell-associated drug (Figure 4A) and found it to be 50% significantly higher in TamC3 (*p* < 0.05) but not in TamR3. However, these changes did not explain the 12-fold increase in drug sensitivity. Flow cytometry, combining measurement of DNA content and of γ-phosphorylation of H2AX as an indication of DNA damage, was used to compare responses to cisplatin and oxaliplatin after 2 h. In comparison to the parental line, both TamC3 and TamR3 showed increased sensitivity to γ-H2AX induction, and increased S-phase arrest (Figure 4B). Increased γ-H2AX induction was found predominantly in S-phase cells (Figure 5) and the increases were consistent with the observed decreases in IC_50_ values (Figure 2).

### Comparison of cellular energy utilization

**Figure 6:**
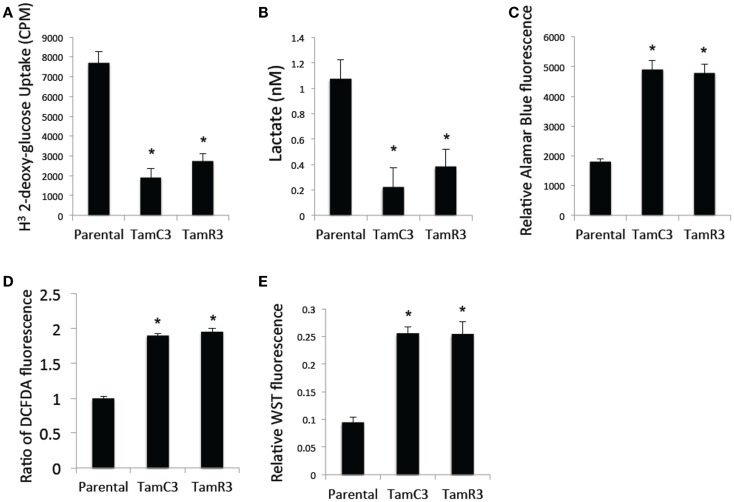
**Comparison of cellular energy utilization**. **(A)** Uptake of 2-deoxy-d-[1-^3^H]glucose by MCF-7 parental, TamC3, and TamR3 cells. **(B)** Lactate concentration in culture medium of MCF-7 parental, TamC3, or TamR3 cells, measured spectrophotometrically. **(C)** Metabolic rate of MCF-7 parental, TamC3, and TamR3 cells, as measured by Alamar Blue assay. **(D)** ROS concentration in MCF-7 parental, TamC3, and TamR3 cells as measured by DCF-DA fluorescence. **(E)** SOD activity in MCF-7 parental, TamC3, and TamR3 cells, as measured by WST assay. *Significantly different from MCF-7 parental line (*p* < 0.05). Results from representative experiments were shown as the mean ± SE from the indicated replicates detailed in the Section “Materials and Methods.”

Since chemoresistance is associated with increased glucose uptake and glycolysis (15–17), we measured glucose uptake using 2-deoxy-d-[1-^3^H]-glucose and found it to be significantly reduced in TamC3 and TamR3 compared to the parental line (Figure 6A). We also measured the lactate concentration in the culture medium as a marker for glycolysis and found it to be significantly lower for TamC3 and TamR3 than for the parental line (Figure 6B). We then determined whether the reduced glucose uptake and glycolysis in TamC3 and TamR3 was accompanied by increased mitochondrial respiration. We used Alamar Blue, an oxidation–reduction sensitive dye that detects oxidation by all the elements of the electron transport chain, as a measure of mitochondrial activity. TamC3 and TamR3 showed increased staining in comparison to the parental line, indicative of increased mitochondrial utilization, in comparison to the parental line (Figure 6C).

Increased mitochondrial activity is associated with increased ROS production (18). We therefore determined cellular ROS concentrations by flow cytometry using the redox-sensitive DCF-DA fluorescent probe; both TamC3 and TamR3 showed almost twice the level of ROS generated by the parental line (Figure 6D). Since cellular production of ROS is generally associated with induction of the intracellular ROS-scavenging system SOD, we a determined cellular SOD content by cleavage of the tetrazolium salt WST-1; this was found to be increased in TamC3 and TamR3 cells as compared to the parental cells (Figure 6E).

**Figure 7:**
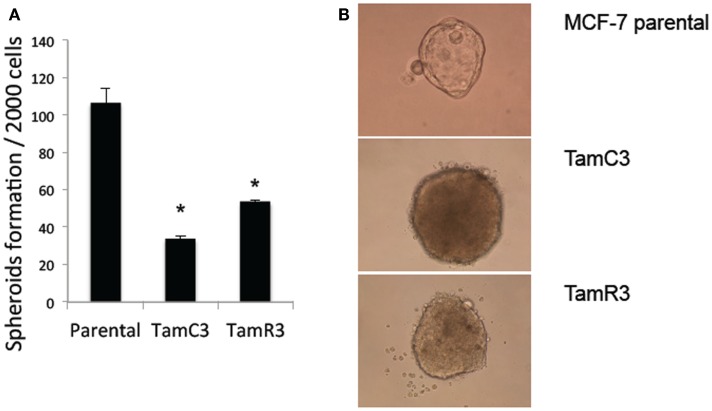
**Mammospheres formation in MCF-7 parental, TamC3, and TamR3 cells**. **(A)** Mammosphere formation efficiency in MCF-7 parental, TamC3, and TamR3 cells. **(B)** Mammospheres morphology of MCF-7 parental (ascini), TamC3, and TamR3 cells (compact spheroid). *Significantly different from MCF-7 parental line (*p* < 0.05).

Stem cell-like properties of breast cancer cells are known to be associated with reduced dependence on mitochondria, as shown by decreased production of ROS and increased glucose metabolism. Since the formation of mammospheres is an indication of stem cell-like character (19, 20), we hypothesized that the increased mitochondrial activity of TamC3 and TamR3 would be associated with a decreased ability to form mammospheres and therefore compared the ability of MCF-7, TamC3, and TamR3 to form mammospheres. As shown in Figure 6A, TamC3 and TamR3 were less efficient than MCF-7 parental cells in mammosphere formation (Figure 7A); they formed only compact spheroids (Figure 7B).

## Discussion

Our results point to the existence of sub-lines of the MCF-7 breast cancer cell line where resistance to 4-hydroxytamoxifen is coupled to increased resistance to inhibitors of the mTOR pathway but increased sensitivity to a variety of cytotoxic anticancer drugs. These properties are consistent with those reported for a number of other hormone-resistant breast cancer lines, where resistance to inhibitors of the mTOR/Akt pathway is generally associated with increased resistance to a broad variety of cytotoxic agents (21). It should be noted, however, that a hormone-resistant triple-negative breast cancer cell line shows increased sensitivity to cisplatin (22).

Increased utilization of the PI3K/mTOR pathway in ER^+^ breast cancer has been associated not only with endocrine resistance but also with resistance to a variety of cytotoxic drugs (23, 24). In contrast, several studies have shown that inhibition of mTOR sensitizes tumor cells to cisplatin, paclitaxel, and doxorubicin (25–28). The latter results are consistent the present results showing decreased utilization of the PI3K/mTOR pathway to be associated with increased sensitivity to cisplatin, paclitaxel, and doxorubicin. To this apparent paradox can be added our more recent finding that continued passage of the TamC3 and TamR3 sub-lines resulted in levels of phosphorylation of p70S6K and Akt that were similar to those of the parental MCF-7 cell line (9). However, these later passages retained increased mitochondria respiration, resistance to mTOR inhibitors, and sensitivity to a variety of cytotoxic agents (unpublished data). Like earlier passages, they also show smaller cell volumes than those of the parental line. One possible interpretation of these results is that the level of Akt and p70S6K phosphorylation does not accurately reflect the degree of utilization of the PI3K/mTOR.

In attempting to find a mechanistic basis for the TamC3/TamR3 phenotype, it should be noted that the TamR3 and TamC3 sub-lines are distinguished from the parental line by their degree of mitochondrial activity; the increased production of reactive oxygen intermediates, as measured by Alamar Blue (NADH/FADH conversion) and WST-1 (superoxide production) assays, is consistent with TamR3 and TamC3 having an increased dependence on mitochondrial metabolism in comparison with the parental line. The reduced 2-deoxyglucose uptake and reduced lactate production also suggest a shift toward increased mitochondrial respiration. The reduced stem cell-like character of TamR3 and TamC3 (Figure 6) also reflects an increased dependence on mitochondrial metabolism, as demonstrated by other groups (19, 20). One factor that could be important in stimulating mitochondrial respiration is the p53 protein (29), which is expressed in the MCF-7 line (30). The p53 pathway is known to link cellular responses to DNA damage, mitotic damage, and oxidative stress to the induction of cell cycle arrest and/or apoptosis (31). It was clear in the studies with cisplatin that the TamC3 and TamR3 sub-lines were much more sensitive than the parental line to the induction increased γ-phosphorylation of H2AX (Figure 4); the ATM kinase is responsible for γ-phosphorylation of H2AX is also responsible activation of p53 (32, 33).

In conclusion, we have demonstrated in MCF-7 cells that long-term deprivation of estrogen or exposure to tamoxifen can lead to the emergence of a hormone-resistant phenotype with dramatically increased sensitivity to a variety of cytotoxic drugs. If clinical hormone-resistant breast cancers with phenotypes similar those described here for TamR3 and TamC3 can be identified, therapies could be based on their increased susceptibility to conventional cytotoxic anticancer drugs.

## Conflict of Interest Statement

The authors declare that the research was conducted in the absence of any commercial or financial relationships that could be construed as a potential conflict of interest.
